# Microbiome-Derived Lipopolysaccharide Enriched in the Perinuclear Region of Alzheimer’s Disease Brain

**DOI:** 10.3389/fimmu.2017.01064

**Published:** 2017-09-04

**Authors:** Yuhai Zhao, Lin Cong, Vivian Jaber, Walter J. Lukiw

**Affiliations:** ^1^Neuroscience Center, Louisiana State University School of Medicine, Louisiana State University Health Sciences Center, New Orleans, LA, United States; ^2^Department of Anatomy and Cell Biology, Louisiana State University School of Medicine, Louisiana State University Health Sciences Center, New Orleans, LA, United States; ^3^Department of Neurology, Shengjing Hospital, China Medical University, Heping District, Shenyang, China; ^4^Department of Neurology, Louisiana State University School of Medicine, Louisiana State University Health Sciences Center, New Orleans, LA, United States; ^5^Department of Ophthalmology, Louisiana State University School of Medicine, Louisiana State University Health Sciences Center, New Orleans, LA, United States

**Keywords:** Alzheimer’s disease, inflammatory degeneration, lipopolysaccharide, microbiome, microRNA, small non-coding RNAs

## Abstract

Abundant clinical, epidemiological, imaging, genetic, molecular, and pathophysiological data together indicate that there occur an unusual inflammatory reaction and a disruption of the innate-immune signaling system in Alzheimer’s disease (AD) brain. Despite many years of intense study, the origin and molecular mechanics of these AD-relevant pathogenic signals are still not well understood. Here, we provide evidence that an intensely pro-inflammatory bacterial lipopolysaccharide (LPS), part of a complex mixture of pro-inflammatory neurotoxins arising from abundant Gram-negative bacilli of the human gastrointestinal (GI) tract, are abundant in AD-affected brain neocortex and hippocampus. For the first time, we provide evidence that LPS immunohistochemical signals appear to aggregate in clumps in the parenchyma in control brains, and in AD, about 75% of anti-LPS signals were clustered around the periphery of DAPI-stained nuclei. As LPS is an abundant secretory product of Gram-negative bacilli resident in the human GI-tract, these observations suggest (i) that a major source of pro-inflammatory signals in AD brain may originate from internally derived noxious exudates of the GI-tract microbiome; (ii) that due to aging, vascular deficits or degenerative disease these neurotoxic molecules may “leak” into the systemic circulation, cerebral vasculature, and on into the brain; and (iii) that this internal source of microbiome-derived neurotoxins may play a particularly strong role in shaping the human immune system and contributing to neural degeneration, particularly in the aging CNS. This “*Perspectives*” paper will further highlight some very recent developments that implicate GI-tract microbiome-derived LPS as an important contributor to inflammatory-neurodegeneration in the AD brain.

## Introduction—Inflammatory Signaling in the Alzheimer’s Disease (AD) Brain

**Figure 1:**
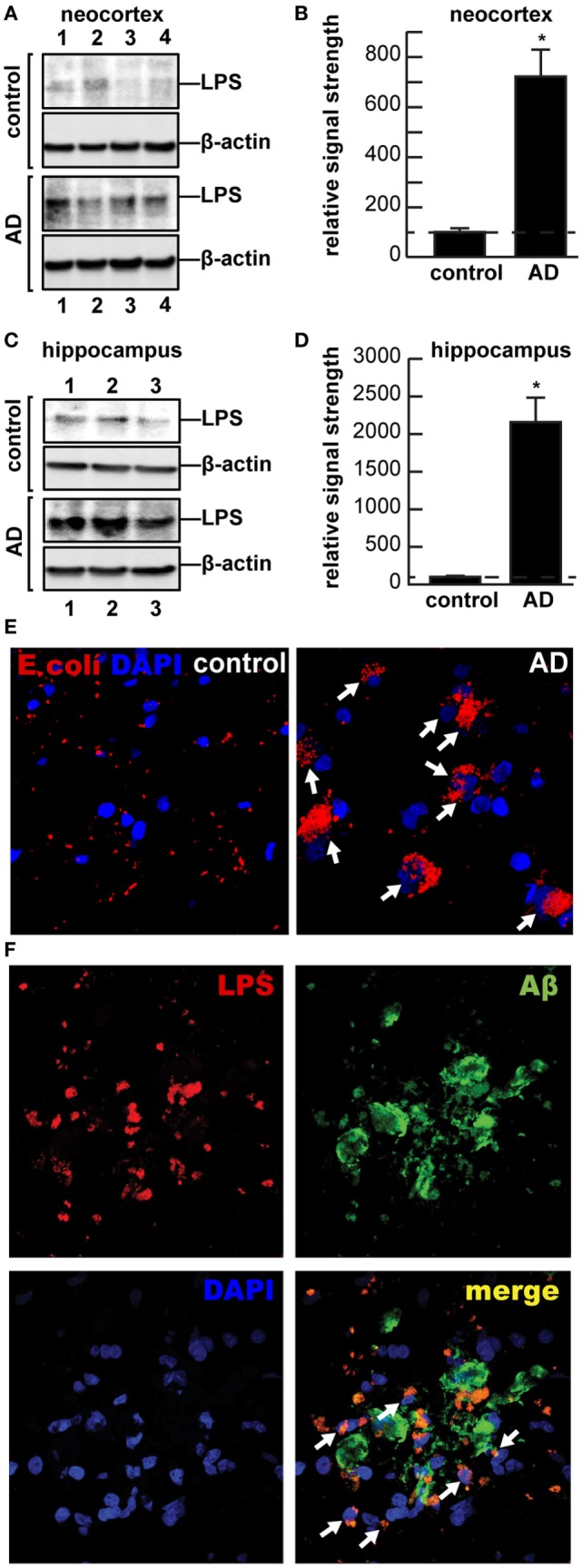
**(A–D)** Western and **(E–F)** immunohistochemical analysis of lipopolysaccharide (LPS) (~37 kDa) signals in human brain temporal lobe neocortex [*N* = 4 control and 4 sporadic Alzheimer’s disease (AD) cases; quantified in **(B)**]; and **(C)** hippocampus [*N* = 3 control and *N* = 3 sporadic AD cases; quantified in **(D)**] were compared against β-actin (~42 kDa) abundance in the same sample (using anti-*Escherichia coli* LPS; cat# ab35654 from Abcam, Cambridge UK and anti-β-actin cat# 3700, Cell Signaling, Danvers, MA, USA). All Western methodologies have been previously described in detail (12, 19). Densitometric readings of immune-reactive bands were obtained using ImageQuantTL [GE Healthcare (12, 19, 20)]; all control and AD tissues were age- and gender-matched; there were no significant differences between the age (control 82.5 ± 8.1 years, AD 81.3 ± 8.8 years), gender (all female), postmortem interval (PMI) (all tissues 3.8 h or less), RNA quality, or RNA yield between each of the two groups; in these samples, LPS abundance was found to be on average greater than sevenfold as abundant in AD when compared to control neocortex; LPS was found to be on average >21-fold as abundant in AD when compared to control hippocampus; in **(B,D)** a dashed horizontal line at 100 is included for ease of comparison; *p < 0.01 (ANOVA); **(E,F)** for immunohisto-chemistry control and AD neocortex and/or hippocampal brain tissues were embedded, sectioned (10 μm), fixed, and incubated with primary antibodies (1:1,000; 1× PBS with 2% BSA, 2% goat or donkey serum, and 0.1% TX-100) overnight at 4°C, washed with PBS, and then incubated with Alexa Fluor-conjugated species-specific secondary antibodies (LPS; red fluorescence λmax ~ 650 nm); sections were next counter-stained with DAPI (blue fluorescence; λmax ~ 470 nm) for nuclei **(E)**, and/or Aβ peptide (green fluorescence; λmax ~ 510 nm) **(F)** and imaged with Zeiss LSM 700 Confocal Laser microscope system (Richmond, VA, USA); note perinuclear staining of LPS in AD; while there appears to be random association of LPS with Aβ deposits in controls, >75% of all LPS signals were found to be associated with brain cell nuclei in AD; the significance of this is not currently known; the association of LPS with the major cellular repository for genetic material suggests that the significance of this association may be genetic; white arrows highlight LPS-nuclear envelope association; a total of 26 control and AD brains (PMI 3.8 h or less) were examined and yielded highly similar results; **(E,F)** magnification 50×.

Multiple aspects of increased inflammatory signaling and an altered innate-immune system are consistent features of AD neuropathology; however, it is not well understood where these pathogenic signals originate or how they progressively contribute to the AD process (1–5). AD is characterized by the appearance of complex networks of many different kinds of chemokines and cytokines including, prominently, interleukin 1β (IL-1β) and tumor necrosis factor (TNFα), 40 and 42 amino acid amyloid beta (Aβ40, Aβ42) peptides, and adhesion molecules, in addition to the progressive deposition of these Aβ peptide containing amyloid plaques and neurofibrillary tangles (NFT) in the parenchyma of AD brain (6, 7). Activated microglia, astrocytes, or neurons appear to mediate the release of these pro-inflammatory molecules and cellular immune components (6, 8–12). Indeed, chemokines, cytokines, the insoluble Aβ42-enriched peptide deposits, NFTs, apoptotic, damaged and vanishing neurons, and activated microglia, and other related pro-inflammatory signals are potent neuropathological stimulants that appear to maintain the AD brain in a “*chronic state of self-reinforcing inflammation”* (2, 7, 10–13). Very recent studies that evaluated the pro-inflammatory potential of several different chemokines, cytokines, Aβ peptides, and lipopolysaccharides (LPS), either alone or in combination, have indicated that when compared, bacterial LPSs exhibit the strongest induction of pro-inflammatory signaling in human neuronal–glial cells in primary coculture of any single inducer, and different LPS extracts from different gastrointestinal (GI)-tract resident Gram-negative bacteria appeared to have different pro-inflammatory potential (12, 14–16). For example, exposure of LPS from the Gram-negative GI-tract abundant *Bacteroides fragilis* to primary human neuronal–glial cells in coculture was found to be an exceptionally powerful inducer of the NF-κB p50/p65 dimer, a known pro-inflammatory transcription factor complex that triggers the expression of pathogenic pathways involved in neurodegenerative inflammation (15, 16). In both neocortex and hippocampus, LPS has been detected to range from a ~7- to ~21-fold increase abundance in AD brain (Figures 1A–D). Along with an avalanche of very recent work from independent laboratories, these observations prompted us to further examine the presence and anatomical location of LPS in AD brains versus age- and gender-matched controls (12, 17, 18).

## Internally Derived Noxious Exudates of the GI-Tract Microbiome

Major Gram-negative bacilli of the human GI-tract, such as the abundant *B. fragilis* and *Escherichia coli* (*E. coli*), are capable of discharging a remarkably complex assortment of pro-inflammatory neurotoxins. These consist of four major components: (i) bacterial amyloids (10, 21); (ii) endotoxins and exotoxins (5, 12); (iii) LPS (12, 18); and (iv) small non-coding RNAs (sncRNAs) [(22–25), unpublished observations]. Either alone or in various combinations, these neurotoxins are intensely pro-inflammatory toward primary human brain cells (12, 15, 16). As integral components of the outer leaflet of the outer membrane of Gram-negative bacteria, LPS shed into the local environment have historically been thought to play some host–pathogen immune-evasion strategy useful to bacterial survival while eliciting strong immune and inflammatory responses within the host. Interestingly, secreted LPS along with proteolytic endotoxins and amyloid monomers are generally soluble as monomers. However, over time, they aggregate into highly insoluble fibrous lipoprotein lesions that associate with the progressive degenerative neuropathology of several common, age-related disorders of the human systemic circulation, and CNS including systemic inflammatory response syndrome, multiple sclerosis, prion disease, and AD (12, 20, 26). LPS, the major molecular component of the outer membrane of Gram-negative bacteria normally serves as a physical barrier providing the bacteria protection from its surroundings. LPS is also recognized by the immune system as a marker for the detection of bacterial pathogen invasion and responsible for the development of inflammatory response is perhaps the most potent stimulator and trigger of inflammation known (27). LPS activates toll-like receptors (TLRs), membrane-spanning protein receptors expressed in microglial cells of the innate-immune system, which recognize common damage- or pathogen-associated molecular-patterns [DAMPS, PAMPs (2, 28)]. Interestingly, of the 13 currently characterized TLRs, the microglial TLR2 and TLR4 are activated by amyloid, LPS, lipoglycans, and/or other microbial triggers that subsequently induce cytokine production, inflammation, phagocytosis, and innate-immune defense responses that directly induce the development of CNS pathology. In addition to the TLR2 and TLR4 receptors, at least one additional microglial transmembrane LPS receptor—CD14 mediates phagocytosis of both bacterial components and Aβ42 peptides, hence expanding roles for microglia and microglial LPS receptors in AD pathophysiology (12, 29).

To cite other recent examples, a secreted, highly pro-inflammatory zinc metalloprotease metalloproteinase *B. fragilis* endotoxin called fragilysin (BFT) derived from enterotoxigenic strains of *B. fragilis* have been recently shown to contribute to: (i) anaerobic bacteremia, sepsis and systemic inflammatory distress, diarrheal disease; (ii) systemic inflammation, GI-tract, and colorectal cancers; (iii) inflammatory neurodegeneration in part *via* the disruption of epithelial cell-based GI-tract barriers *via* cleavage of the synaptic adhesion zonula adherens protein E-cadherin; and (iv) enterotoxigenic microbes specifically impact microglial-mediated innate-immune responses, detoxifying and phagocytic mechanisms, and amyloidogenesis characteristic of inflammatory aspects of neurodegeneration (12, 15, 16, 30–34). Prokaryotic sncRNAs play essential roles in the regulation of many bacteriological processes including the expression of exotoxins and endotoxins and the regulation of bacterial virulence (22). In eukaryotes, microRNAs (miRNAs) also function as key regulators in many biological processes through posttranscriptional suppression of mRNAs and the downregulation of gene expression. Typical trans-acting microRNA-size sncRNAs are abundant in all prokaryotic cells including bacteria and fungi, but their production, release, and leakage from the confines of a healthy GI-tract into systemic and cerebral circulation and downstream effects along the gut microbiome–brain axis are a highly novel and largely unexplored research area (12, 22, 25). There is considerable speculation that, as for other bacterial exudates, such RNA-based neurotoxins may be pathogenic and highly detrimental to the homeostatic function of the neuronal, glial, endothelial, and other brain cells that comprise the CNS (23, 24).

## Leakage of Neurotoxic Molecules into the Systemic Circulation and the CNS

Gram-negative bacterial exudates of the human GI-tract are not only the primary source of a remarkable array of neurotoxic pro-inflammatory amyloids, endo- and exotoxins, LPSs, and sncRNAs but also serve as potent sources of membrane-disrupting agents (12, 15, 16, 35, 36). As aforementioned, BFT can alone induce the disruption of epithelial cell-based GI-tract membrane barriers *via* presenilin 1-dependent cleavage of the zonula adherens protein E-cadherin, thus leading to progressive functional decline in membrane integrity (12, 15, 16, 30–34). Other recent reports suggest that intestinal dysbiosis and “*leaky gut syndrome*” constitutes a key pathophysiological link for transport of microbiome-derived toxins across GI-tract and blood–brain biological barriers that result in a progression from systemic to CNS inflammation (12, 21). The progressive failure of major physiological barriers is reminiscent of the activation of the thanatomicrobiome (*the “death”-associated microbiome*) and the deactivation of protective biological barriers that occurs at the time of death when normal endothelial cell structures and signaling: (i) becomes increasingly inoperative and “*leaky*” (1, 12, 37); and (ii) progressively unable to support normal homeostatic brain functions that are accompanied by a progressive and insidious functional decline (12, 28, 37). These recent findings indicate that AD-affected brains have remarkably large loads of bacterial-derived toxins compared to controls. The transfer of noxious, pro-inflammatory molecules from the GI-tract microbiome to the CNS may be increasingly important during the course of aging when both the GI-tract and blood–brain barriers become significantly more permeable (12, 28, 38).

## Perinuclear Localization of LPS in AD Brains

**Figure 2:**
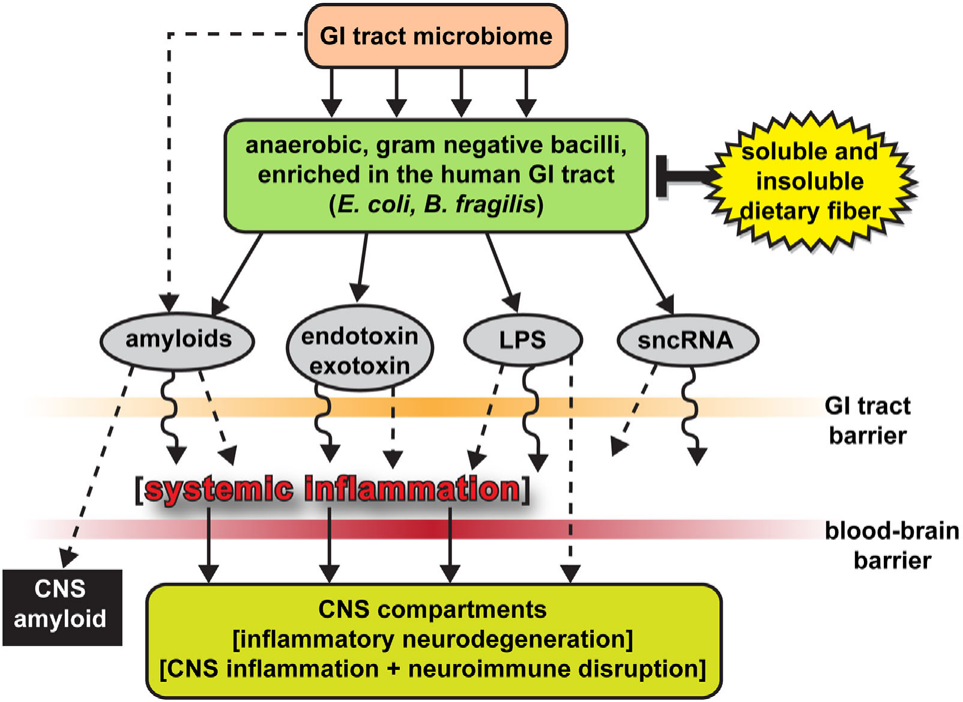
The human gastrointestinal (GI)-tract microbiome as a source of strong pro-inflammatory exudates—highly schematicized depiction of anaerobic, Gram-negative bacilli (such as *Escherichia coli* and *Bacteroides fragilis*) of the human GI-tract microbiome and their potentially pathogenic, immunogenic, and pro-inflammatory neurotoxins [amyloids, endotoxins and exotoxins, lipopolysaccharide (LPS), and small non-coding RNAs (sncRNAs)] that may contribute to systemic and CNS inflammation and neuro-immune disruption; two major sources of these complex mixtures are *E. coli* and *B. fragilis*; major anaerobic Gram-negative bacilli of the human middle and lower GI-tract, respectively; the *B. fragilis* toxin (BFT) fragilysin is one of the most potent pro-inflammatory molecules known (12, 15, 16, 30, 37, 38); these intensely pro-inflammatory LPS species may be able to “leak” through at least two major biophysiological barriers—the GI-tract barrier and the blood–brain barrier—to access brain compartments [see Ref. (2, 12, 28, 30, 31, 34)]. Neurotoxic mixtures secreted by multiple GI-tract microbes or other microbial species may have considerable potential to support inflammatory signaling within the CNS (2, 12, 21, 28, 30, 31, 34); *B. fragilis* proliferation and (BFT) fragilysin levels may be kept in check by increased intake of soluble and insoluble dietary fiber (34, 38, 46); interestingly, BFT-derived fragilysin may exert neurotoxic activities *via* multiple mechanisms: (i) by increasing the permeability or “leakiness” of the intestinal epithelium *via* the dissolution of tight junctions in epithelial cells (28, 30); and (ii) by promoting amyloid peptide aggregation and progressive amyloidogenesis (15, 16, 18, 37, 38); Figure 2 modified and updated from Lukiw (15, 16).

While other recent studies have reported an LPS-mediated stimulation of chronic inflammation, beta-amyloid accumulation, and episodic memory decline in murine models of AD (39, 40) and a biophysical association of LPS with amyloid deposits and blood vessels in human AD patients (18), here, we provide the first evidence of a perinuclear association of LPS with AD brain cell nuclei (Figures 1E,F). Strong adherence of LPS to the nuclear periphery has recently been shown to inhibit nuclear maturation and function that may impair or block export of mRNA signals from brain cell nuclei, a highly active organelle with extremely high rates of transcription, mRNA processing, and export into the cytoplasm [(41–43), unpublished observations]. This may in part be responsible for the widely observed, generalized downregulation of global gene expression in AD, independently reported by several AD gene expression research laboratories, through the biophysical blockage of mRNA trafficking through nuclear pores (41, 42, 44, 45). LPS may be further injurious to the nuclear membrane just as LPS contributes to cerebrovascular endothelial cell membrane injury (12, 18, 40). Lastly, evidence is accumulating that neurotoxic exudates from other GI-tract microbiota may contribute to dysfunction in additional, ultimately fatal neuropsychiatric illnesses that involve progressive inflammatory neurodegeneration (8, 12). New opportunities to modulate existing gut microbiota and their exudates using probiotics and/or modifications through soluble or insoluble dietary fiber intake could provide novel targets for more effective clinical intervention [Figure 2 (18, 46, 47); unpublished observations]. Interestingly, the high intake of dietary fiber is a strong inhibitor of *B. fragilis* abundance and proliferation in the intact human GI-tract and as such is a potent inhibitor of the neurotoxic *B. fragilis*-derived amyloids, LPS, enterotoxins, and sncRNAs. Hence, dietary fiber-mediated suppression of *B. fragilis* abundance may turn out to be beneficial for *both* the human GI-tract microbiome and CNS health (34, 38, 46).

## Concluding Remarks

It is not generally appreciated that, in the human body, microbial genes outnumber human genes by about 100 to 1, and the impact of bacterial genetics on human health and disease may have been vastly underestimated (8, 12, 15–17, 48). The assumption of the privileged immunological status of the CNS has also been recently questioned in multiple investigations, particularly in terms of inflammatory neurodegenerative diseases such as AD, as both microbial-derived nucleic acid sequences and/or noxious exudates representative of GI-tract Gram-negative bacteria are showing up within CNS compartments, including, prominently, anatomical regions of the CNS involved in inflammatory and pathological signaling and neuro-immune disruptions that characterize the AD process (9, 12, 15, 16, 18, 49). For example, LPS has been recently localized to the same anatomical regions involved in AD-type neuropathology to levels of greater than sevenfold over control in the temporal lobe neocortex and >21-fold over control in the hippocampus. This suggests that GI-tract microbiome-derived LPS may be an important initiator and/or significant contributor to inflammatory degeneration in the AD CNS (Figures 1 and 2). An alternative, yet, highly speculative view is that the human CNS may have its own microbiome, which could also explain the presence of Gram-negative bacterial secretory components in the brain as well as multiple forms of microbial-derived nucleic acid sequences (12, 49).

## Author Contributions

YZ, LC, VJ, and WL conceived and discussed the experimental design; YZ, LC, VJ, and WL performed the experiments; YZ and WL performed bioinformatics and contributed to the medical artwork; WL reviewed the results and further researched and wrote this paper.

## Conflict of Interest Statement

The authors declare that the research was conducted in the absence of any commercial or financial relationships that could be construed as a potential conflict of interest.
